# Analysing celebrity and influencer marketing of food and beverages to adolescents on Instagram

**DOI:** 10.1017/S1368980025101006

**Published:** 2025-08-27

**Authors:** Rida Khan, Afifa Tanweer, L Suzanne Suggs

**Affiliations:** 1 Institute of Communication and Public Policy, Faculty of Communication, Culture and Society, University of Lugano, Lugano, Switzerland; 2 Department of Nutrition and Dietetics, School of Health Sciences, University of Management and Technology, Lahore, Pakistan

**Keywords:** Teenagers, Instagram, Food, Advertisement, Marketing, Social Media, Digital

## Abstract

**Objective:**

To assess the nutritional quality of foods and beverages (F&B) advertised to adolescents and analyse marketing techniques and persuasive appeals used by celebrities and influencers on Instagram.

**Design:**

A content analysis study was conducted using the WHO’s CLICK Monitoring Framework and Nutrient Profile Model.

**Setting:**

Instagram, a popular social media platform among adolescents with frequent F&B advertisements by celebrities and influencers.

**Participants:**

The top forty-eight Instagram accounts of celebrities and influencers posting F&B advertisements were selected based on follower count and engagement metrics. Nutrient profiling of advertised F&B (*n* 344) and content analysis of posts featuring F&B (*n* 326) between January 2021 and May 2023 were performed. Data collected included characteristics of celebrities and influencers, marketing techniques, online engagement and persuasive appeals in the posts.

**Results:**

Carbonated beverages and flavored waters (28·5 %), energy drinks (20·6 %) and ready-made foods (15·4 %) were most frequently advertised, with the majority (89·2 %) of products not permitted for advertisement to adolescents, according to WHO. Common marketing techniques included tagging brand (96·9 %) and using brand logo (94·2 %). The most frequently used persuasive appeals were taste (20·9 %), energy (10·7 %), link to sports events (10·7 %), new product (9·5 %) and fun (7·4 %).

**Conclusion:**

Most F&B advertised on Instagram by celebrities and influencers are prohibited from being advertised to adolescents by the WHO. This highlights the need for stricter regulation of user-generated content and for users and parents to be better educated about persuasive techniques used on social media to make them less vulnerable to the influence of marketing.

Digital media provides a landscape for the food and beverage (F&B) industries to advertise and promote their products online, often for free. Digital marketing on social media has become a cost-effective strategy adopted by the F&B industry because traditional media is becoming less popular among the younger generations^(1)^, and digital media is less regulated. Chile and Portugal are the only two countries that regulate F&B advertisements on digital media^(2)^. There are currently 4·9 billion social media users worldwide, and the number is forecasted to increase to 5·85 billion in 2027^(3)^. Adolescents are among the most active users of smartphones, the Internet and social media^(4)^. Common Media Sense, a non-profit organisation in the USA, reported in 2021 that 38 % of adolescents aged 13 to 18 years use social media compared with 31 % in 2019^(5)^. In 2023, almost half of the USA teens reported using social media^(5)^. TikTok (63 %), Snapchat (60 %) and Instagram (59 %) are the most popular social media sites for teens in the USA^(6)^.

Celebrities and social media influencers can provide the stimulus for the purchase and consumption decisions of their online followers^(7)^. These celebrities and influencers often include actors, singers, sportspersons, social media personalities and online content creators with substantial online followings^(8)^. F&B companies utilise the popularity and large fan base of such celebrities and influencers to promote their brand or products through a paid partnership or gift endorsement, but the distinction between the advertisement and personal content can be easily blurred^(9)^. Food advertisements by celebrities and influencers have been shown to enhance the recall of advertisements and persuasiveness among children and adolescents^(10)^. Research has shown that F&B industries utilise social media not only to send influential marketing messages but also to encourage active communication, engagement and interaction^(11)^. Furthermore, these marketing strategies are especially designed to appeal to teenagers, thereby increasing their effectiveness^(12)^.

The WHO states that the effectiveness of digital food marketing relies on the exposure and power of the communication message^(13)^. Exposure refers to the reach and frequency of the marketing message, while power refers to the content of the message and the creative marketing strategies used to make the marketing message persuasive to consumers^(13,14)^. A recent study by Neito *et al.* (2023) using real-time screen capture data from Mexican children and adolescents found that almost 70 % of them were exposed to food marketing on digital media, and brand characters were the most frequently used marketing technique^(15)^. Although social media applications have an age restriction of 13 years old or above, the researchers found that many children and adolescents were using social media applications despite the age restriction^(15)^. In a randomised control trial, Bragg *et al.* (2021) concluded that the artistic appearance of Instagram advertisements makes it difficult for teenagers to perceive social media ads as marketing^(16)^. Food industries and celebrities on social media use symbolic relevance purposively directed towards adolescents who are most vulnerable to the advertisement puffery^(17)^. They use different creative attention-getting techniques, such as animation, music, singing, rhyming and dancing, to ensure that kids and teenagers pay attention to the ads^(18,19)^. According to Elliott *et al.* (2024), adolescents identify persuasive creative content, such as visual style, theme, special offer and humour, as the top persuasive appeals in social media advertisements^(20)^.

According to the Social Cognitive Theory^(21)^, the liking of a particular individual may lead to imitation of their actions and preferences. Accordingly, teenagers are more likely to purchase or consume F&B from a particular brand that they see posted by celebrities and influencers whom they follow^(22)^. Furthermore, persuasive appeals of advertisements on social media may weaken the cognitive defenses in teens, making them more vulnerable to the influence of marketing^(23)^. Research in consumer behaviour, marketing and advertising has incorporated the sociological phenomena of symbolic interaction to explain the most effective strategies for food marketers^(24)^. Therefore, the objective of the research was to understand the nutritional quality of foods promoted by celebrities and social media influencers, the level of online social interaction and engagement with them and which marketing techniques and persuasive appeals are used in F&B promotion.

## Methods

**Fig. 1:**
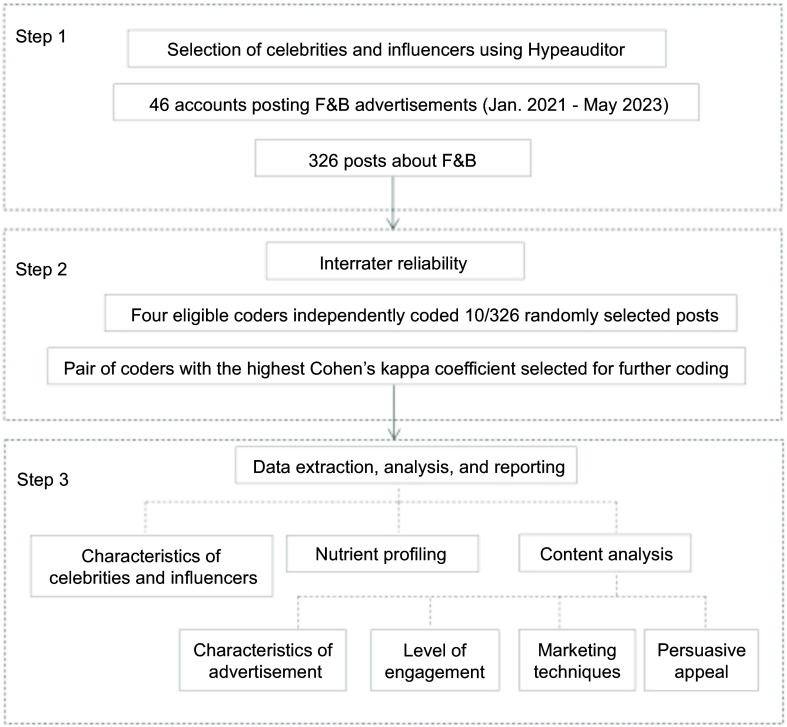
Summary of the methods to analyse food and beverage marketing by celebrities and influencers on Instagram.

A content analysis study was conducted based on the WHO Regional Office for Europe CLICK Monitoring Framework^(25)^. This framework provides a comprehensive method for the analysis of F&B marketing to adolescents on social media. Instagram was selected for this study because it is one of the most popular social media platforms among adolescents^(5)^. The platform’s visual and interactive features enable celebrities and influencers to engage with their followers^(26)^. Furthermore, Instagram boasts a higher engagement rate between advertisers and users as compared with other platforms^(27)^. Pre-established coding of the variables to analyse the marketing technique, persuasive appeals and online engagement developed by the WHO regional office for Europe was used for the content analysis^(28)^. A summary of methods is given in Fig. 1.

### Sample selection

HypeAuditor, a data-driven analytics platform, was used to select celebrities and influencers for our study. HypeAuditor employs a ranking system that assesses Instagram users based on their genuine follower count and engagement metrics, including likes and comments from real Instagram users, which are regularly updated. A list of the top 1000 Instagram influencers in May 2023 was used to select the accounts for this study^(29)^. The reason for selecting accounts with the highest followers was to analyse the content with maximum audience reach. Based on previous studies using content analysis of social media posts, a target sample size of approximately 300 posts was set to ensure sufficient analytical depth and thematic saturation without overextending the search process^(30,31)^. First, the Instagram accounts of the top 100 influencers were searched for F&B brand or product marketing in the last 2 years. Later, the search scope was gradually expanded to additional accounts until relevant content could be identified, and a final cut-off was set at 180 accounts, at which point a sufficient number (*n* 326) of F&B-related posts had been collected. Oversampling beyond the initial target of 300 posts was to account for potential data loss and because certain influencers had a high volume of relevant posts. Details about the accounts, including the account name and the number of followers, were recorded. Of the top 180 accounts, forty-six users generated content related to F&B advertisements between January 2021 and May 2023. This time frame was selected to ensure the sample reflected recent trends and captured posts across different seasons, such as football championships, festivals and holidays. A total of 326 F&B posts were found. Each post was assigned a unique item number for analysis.

### Coder training and interrater reliability

Protocols and templates for monitoring food marketing on Instagram were obtained from the Special Initiative on Noncommunicable Diseases and the Innovation Unit of the WHO regional office for Europe^(28)^. The protocols and templates were used to train four coders who were final-year students in the Bachelor’s program in nutrition sciences at the University of Management and Technology to perform the content analysis and nutrient profiling under the supervision of the research coordinators. The coding guide, adapted from the template of the WHO, is given in Table 1. The researcher read the protocol with the coders, explained in detail wherever necessary and coded ten sample posts in the templates.

**Table 1.** Coding guide to analyse food and beverage marketing by celebrities and influencers on Instagram

Codes	Name of code	Subcodes	Names of subcodes	Measure/data
1	Characteristics of influencers	1·1	Real name	String
		1·2	Instagram handle	String
		1·3	Followers	Numeric-discrete
		1·4	Gender	Numeric-discrete (binary-Male/Female)
		1·5	Profile link	String
		1·6	Post link	String
		1·7	Country of account	String
		1·8	Posts advertising F&B	Numeric-discrete
		1·9	Date of post	String
2	Nutrient profiling	2·1	Product classification	Numeric-discrete (22 sub categories (17 for food, 5 for beverages) (WHO Nutrient Profile Model 2023)
		2·2	Nutrient profiling^*^	Numeric-discrete (binary-Permitted^†^/Not permitted)
3	Content Analysis	3·1	Characteristics of advertisement	–
		3·1·1	Presence of brand name	Numeric-discrete (binary-Yes/No)
		3·1·2	Brand status	Numeric-discrete (binary-Product brand/Food retail enterprise)
		3·1·3	Product name	Numeric-discrete (binary-Present/Absent)
		3·1·4	Number of products in a post	Numeric-discrete (No product shown/ Single/ Multiple)
		3·1·5	Focus of post	Numeric-discrete (binary-Brand/Product)
		3·2	Marketing techniques	–
		3·2·1	Type of post	Numeric-discrete (binary-Image/Video)
		3·2·2	Added sound	Numeric-discrete (binary-Yes/No)
		3·2·3	Reason for featuring brand/product	Numeric-discrete (paid endorsement/gift endorsement/influencer’s own brand/no explicit indication)
		3·2·4	Location of food shown in the post	Numeric-discrete (food retail enterprise/supermarket/home/entertainment event/outdoors/gym or sports event/digital background)
		3·2·5	Brand logo	Numeric-discrete (binary-Present/Absent)
		3·2·6	Brand tagged in the post	Numeric-discrete (binary-Yes/No)
		3·2·7	Image of packaging	Numeric-discrete (binary-Present/Absent)
		3·2·8	Image of the product	Numeric-discrete (binary-Present/Absent)
		3·2·9	Brand equity characters, licensed characters and other celebrity endorsers	Numeric-discrete (binary-Present/Absent)
		3·2·10	Health claims (sugar-free/ no added sugar, healthy food, provides essential nutrients)	Numeric-discrete (binary-Present/Absent)
		3·2·11	Health washing (false impression that the product is healthy)	Numeric-discrete (binary-Present/Absent)
		3·2·12	Physical activity shown	Numeric-discrete (binary-Yes/No)
		3·2·13	Presence or link to an entertainment event, sports event, special day	Numeric-discrete (binary-Yes/No)
		3·2·14	Premium offers	Numeric-discrete (binary-competition/not competition)
		3·2·15	Prompt to engage with the post online	Numeric-discrete (following/liking/sharing/commenting/tagging/posting text or video)
		3·3	Post engagement	–
		3·3·1	Likes	Numeric-discrete
		3·3·2	Comments	Numeric-discrete
		3·4	Persuasive appeal	Numeric-discrete^‡^ (quantity/convenience/taste/health/nutrition/ energy/price/uniqueness/fun/family relationships/general superiority/peer status/friendship/romance/sex appeal/premium offer/contest/weight loss/diet, offers choices/options/ enjoyment/satisfaction/new product introduction/corporate information/humor/magic/fantasy/link to event or entertainment/ holiday/travel or adventure/novel or surprising feature/corporate promotion)

* Comparison against the respective threshold for each of the following nutrients: i. Total fat, ii. saturated fats, iii. total sugars, iv. added sugars, v. Na, vi. non-sugar sweeteners.

† Not permitted if it exceeds the threshold.

‡ Top five frequently used appeals in the sample were selected.

After training, ten randomly selected posts from the sample of 326 were allocated to four coders for the independent content analysis. Queries from the coders were gathered and resolved by the researcher. Statistical coefficient Kappa was calculated by using the Statistical Package for Social Sciences version 22 to determine interrater reliability. Content analysis of randomly selected posts from each coder was compared with the others. Six pairs of comparisons were generated, and the pair with the highest Cohen’s kappa coefficient (0·735, *P* value < 0·001) was chosen for the content analysis and nutrient profiling of the sample. A Cohen’s kappa coefficient between 0·61 and 0·8 indicates substantial agreement^(32)^, making the pair of coders most suitable and reliable for this study. Any discrepancies during the data collection were discussed and resolved with the researcher.

### Data collection

Data regarding the characteristics of influencers, nutritional quality of the F&B in the post and the contents of posts were collected and coded by the selected coders.

#### Characteristics of celebrities and influencers

The following information was collected as the characteristics of selected celebrities and influencers on Instagram: real name of the celebrity or influencer, Instagram handle, number of followers, gender, profile link, post link, country of account and number of posts advertising F&B. The dates of posts were also recorded.

#### Product categorisation and nutrient profiling

WHO Nutrient Profile Model (NPM) 2023^(33)^ was used for the nutrient profiling of the F&B advertised in the posts. Following WHO NPM 2023, alcoholic beverages, breast milk substitutes, complementary foods, food supplements and food for special dietary uses were excluded from the scope of this study. F&B shown in the posts were classified into eighteen categories. Different F&B advertised in the same post were categorised and analysed separately. Different flavours of the same product appearing in the same post were analysed together. Websites of the F&B companies were searched to find the nutrient labelling of the product. From the label, the amount of total fat, saturated fat, total sugars, added sugars, non-sugar sweeteners, Na, energy and fibre per 100 grams of food or beverage was calculated. Then, it was compared with the cut-off values and guidelines of the WHO NPM 2023 to establish whether the advertisement of that product is permitted or not.

### Content analysis

Coders logged into their Instagram accounts on their mobile phone devices and opened the sample post links. Contents of the posts were coded into the following four sections using the pre-established codebook^(28)^:

#### Characteristics of advertisement

To capture the general characteristics of advertisement, variables such as the presence of brand name, brand status (product brand, i.e. brand features F&B products or food retail enterprises brand), presence of product name, number of products in a post (no product shown, single, multiple) and focus of post (brand focus rather than product focus) were used.

#### Marketing techniques

Following main marketing techniques were included in this research: Type of post (image or video); added sound; reason for featuring brand/product (paid endorsement, gift endorsement, influencer’s own brand and no explicit indication); location of food shown in the post (food retail enterprise, supermarket, home, entertainment event, outdoors, gym or sports event and digital background); the presence of the brand logo; brand tagged in the post; the image of packaging; the image of the product; the presence of brand equity characters, licensed characters and other celebrity endorsers; the presence of health claims (sugar-free/ no added sugar, healthy food, provides essential nutrients); health washing (false impression that the product is healthy); physical activity shown; presence or link to an entertainment event, sports event, special day; premium offers (competition, not competition); engagement (prompt to engage with the post online by following, liking, sharing, commenting, tagging, posting text or video)

#### Level of engagement

The level of social interaction and online engagement was estimated by the number of likes and comments on the posts.

#### Persuasive appeal

A post may contain several marketing techniques, but the primary message that the influencers use to try to convince or persuade adolescents to purchase the product is the main persuasive appeal of the post^(20,28)^. Persuasive appeals were identified based on the complete content of each post, including visuals, captions and audio (when applicable). A list of persuasive appeals was prepared based on previous studies^(9,15,20)^ and the template provided by WHO^(28)^. The persuasive appeal of the post was selected from the list including quantity, convenience, taste, health/nutrition, energy, price, uniqueness, fun, family relationships, general superiority, peer status, friendship, romance/sex appeal, premium offer/contest, weight loss/diet, offers choices/options, enjoyment/satisfaction, new product introduction, corporate information, humor, magic/fantasy, link to a special day or event or entertainment, holiday, travel or adventure, novel or surprising feature and corporate promotion. After identifying the persuasive appeal of each post, the five most frequently used appeals were selected for case study analysis. The sample posts for each appeal were reviewed to identify typical examples, and snapshots of the posts (images and video) were taken for comparison. A total of five Instagram posts, one for each persuasive appeal, were selected to illustrate how the appeal appears in influencer posts.

### Statistical analysis

The descriptive analyses were conducted using SPSS version 22. Data for the categorical variables are presented as frequencies and percentages. Data for the number of likes and comments were checked for normality by the Shapiro–Wilk test. Data were not normally distributed; hence, the median and inter-quartile range (25^th^ and 75^th^ quartile) were used for analysis.

## Results

### Characteristics of selected celebrities and influencers

Forty-six influencers posted content related to F&B marketing (Table 2). Half of the influencers were male. Male influencers posted more frequently than female influencers (56·1 % *v*. 43·9 %). Thirty-one accounts belonged to entertainment celebrities, eight to sports celebrities and seven to social media influencers. The number of followers ranged from 495 million for the sportsperson Lionel Messi to 13·3 million for the Bollywood actor Yash. The total number of posts by each influencer containing F&B advertisements during the study period ranged from 1 to 35. Sixteen accounts were based in India, eleven in the USA and a smaller number of accounts were distributed across the United Kingdom (*n* 2), Italy (*n* 1), Spain (*n* 2), Brazil (*n* 4), Mexico (*n* 1), South Korea (*n* 1), Australia (*n* 1) and Turkey (*n* 1). For six accounts, the location of the celebrity or influencer was not shared publicly.

**Table 2:** Characteristics of celebrities and influencers on Instagram

Influencer name	Instagram handle	Gender	Country of account	No. of followers	No. of posts advertising food and beverages
Lionel Messi	leomessi	Male	Italy	495M	18
Dwayne Johnson	therock	Male	USA	398M	35
Justin Bieber	justinbieber	Male	USA	292M	8
Virat Kohli	virat.kohli	Male	India	265M	21
Jenifer Lopez	jlo	Female	USA	253M	3
Kourtney Kardashian	kourtneykardash	Female	USA	224M	2
Neymar	neymarjr	Male	Brazil	217M	11
Cardi B	iamcardib	Female	USA	169M	3
Gal Gadot	gal_gadot	Female	–^*^	109M	11
Lisa	lalalalisa_m	Female	–^*^	99·9M	1
Shraddha	shraddhakapoor	Female	India	85·2M	11
Gigi Hadid	gigihadid	Female	USA	78·9M	1
Jisoo	sooyaaa__	Female	South Korea	76·7M	1
Katrina Kaif	katrinakaif	Female	India	79·2M	2
Camila	camila_cabello	Female	USA	66·9M	5
Jacqueline Fernandez	jacquelienefernandez	Female	India	68·8M	12
Akshay Kumar	akshaykumar	Male	India	67·1M	2
Anushka Sharma	anushkasharma	Female	India	66·9M	7
Mohamed Salah	mosalah	Male	UK	63·3M	3
Paul Labile Pogba	paulpogba	Male	–^*^	61·5M	9
Isabella Khair Hadid	bellahadid	Female	USA	61M	21
Whinderssonnunes	whinderssonnunes	Male	Brazil	59·7M	3
Chris Hemsworth	chrishemsworth	Male	Australia	58·5M	1
Jannat Zubair Rahmani	jannatzubair29	Female	India	48·1M	2
Jimmy Donaldson	mrbeast	Male	US	46·8M	6
Varun Dhawan	varundvn	Female	India	46·8M	3
Hrithik Roshan	hrithikroshan	Male	–^*^	46·6M	5
Shah Rukh Khan	iamsrk	Male	India	45·1M	4
Virginia Fonseca Costa	virginia	Female	Brazil	44·7M	1
Blake Lively	blakelively	Female	UK	43·6M	11
Vinícius José Paixão de Oliveira Júnior	vinijr	Male	Spain	43·6M	4
Rashmika Mandanna	rashmika_mandanna	Female	India	40·7M	13
Addison Rae	addisonraee	Female	USA	36·6M	1
Luka Modrić	lukamodric10	Male	Spain	33·7M	1
Luisillo El Pillo	luisitocominica	Male	Mexico	33·6M	1
Alia Advani	kiaraaliadvani	Female	India	33M	4
Kartik Aaryan	kartikaryan	Male	India	31·8M	27
Viih Tube	viihtube	Female	Brazil	31·5M	17
Hande Ercel	handemiyy	Female	Turkey	31·4M	2
Samantha	samantharuthprabhuoffl	Female	India	31·1M	12
Hardik Himanshu Pandya	hardikpandya93	Male	India	28·7M	9
Noah Schnapp	noahschnapp	Male	USA	24·6M	4
BZRP	bizarrp	Male	–^*^	21M	1
Vijay Deverakonda	Thedeverakonda	Male	India	20·4M	4
Auron	auronplay	Male	–^*^	18·5M	2
Yash	thenameisyash	Male	India	13·3M	1

* Location of the account not shared.

### Product categorisation and nutrient profiling

Twenty-nine of the 326 posts featured multiple F&B products, so the total sample for nutrient profiling became 344 products. The most frequent F&B products advertised were carbonated beverages and flavoured waters, making up 28·5 % of all the products (see Table 3). Energy drinks were the second most advertised product, with almost one-fifth (20·6 %) of the total posts. The third most frequently advertised products were from the category of ready-made, convenience foods and composite dishes (15·4 %). Next were chocolates and sugar confectioneries (11 %) and savory snacks (8·1 %). Cakes, sweet biscuits, pastries or other bakery products made up 5·5 % of posts, while 4·8 % of the products were plant-based food.

**Table 3:** Product categories and nutrient profiling of the food and beverages advertised in the posts of celebrities and influencers on Instagram

Product categories^*^	Frequency (*n* 344)	Percentage
Soft drinks, bottled waters and other drinks	98	28·5
Energy drinks	71	20·6
Ready-made and convenience foods and composite dishes	53	15·4
Chocolate and sugar confectionery, energy bars, sweet toppings and desserts	38	11·0
Savory snacks	28	8·1
Cakes, sweet biscuits, and pastries; other sweet bakery wares and dry mixes for making such	19	5·5
Savory plant-based foods/ meat analogues	17	4·8
Edible ices	9	2·6
Dairy milk drinks	8	2·3
Number of excess critical nutrients		
Not permitted to market at all	137	39·8
1 nutrient	114	33·1
2 nutrients	36	10·5
3 nutrients	9	2·6
4 nutrients	11	3·2
Excess critical nutrients		
Added sugar	191	55·5
Caffeine	70	20·3
Non-sugar sweetener	59	17·2
Fat	37	10·8
Calories	34	9·9
Salt	25	7·3
Saturated fat	13	3·8
Total sugar	5	1·5
Marketing permission according to WHO NPM 2023		
Permitted	30	8·7
Not permitted	307	89·2
Could not be determined due to lack of relevant nutritional information	7	2

* Product categories 6: breakfast cereals and 18: sauces, dips and dressings with a frequency of < 2 % were excluded from the table.

Among the products analysed, 39·8 % were not permitted to market at all according to the WHO NPM 2023^(33)^, regardless of the nutrient profile. This includes chocolate and sugar confectionery, energy bars, sweet toppings and desserts; cakes, sweet biscuits and pastries; other sweet bakery wares and dry mixes for making such energy drinks and edible ices. Products with at least one critical nutrient (total fat, saturated fat, total sugars, added sugar, non-sugar sweeteners, salt, caffeine and total calories)^(33)^ in excess accounted for 33·1 % of posts, 10·5 % of products had two critical nutrients in excess, 2·6 % of products had three critical nutrients and 3·2 % of products had up to four critical nutrients in excess.

Added sugar was in 55·5 % of products, 20·3 % had added caffeine and 17·2 % had non-sugar sweeteners. Any amount of added sugar, caffeine and non-sugar sweetener in a product is considered excess and is not permitted to be advertised, according to WHO NPM 2023. Excess fat was found in 10·8 % of products, 9·9 % had excess calories, 7·3 % had excess salt, 3·8 % had excess saturated fat and 1·5 % had excess total sugar.

According to the WHO NPM 2023, 89·2 % of products were not permitted to be advertised/marketed to adolescents, while 8·7 % were permitted.

### Content analysis

A summary of the content analysis of the F&B advertisement by celebrities and influencers on Instagram is presented in Table 4.

**Table 4:** Content analysis of the selected food and beverage posts of celebrities and influencers on Instagram

	Frequency (*n* 326)	Percentage
Characteristics of advertisements		
Brand name present	326	100
Brand status		
Product brand	290	89
Food retail enterprise brand	36	11
Product name present	292	89·6
Number of products in a post		
No product shown	33	10·1
Single	264	81
Multiple	29	8·9
Brand focus rather than product focus	36	11
Marketing techniques		
Type of post		
Image	113	34·7
Video	213	65·3
Added sound feature	183	56·1
Reason for featuring brand/product		
Paid endorsement	126	40·2
Gift endorsement	1	0·3
Influencer’s own brand	68	20·9
No explicit indication	131	40·2
Brand equity characters	5	1·5
Licensed characters	1	0·3
Other influencer/celebrity	63	19·3
Brand logo present	307	94·2
Brand tagged in post	316	96·9
Image of packaging	274	84
Image of product	194	59·5
Location of food shown in post		
Food retail enterprise	23	7·1
Supermarket	10	3·1
Home	116	35·6
Entertainment event	27	8·3
Outdoors	68	20·9
Gym or sports event	38	11·7
Digital background	44	13·5
Health washing	81	24·8
Health claims	74	22·7
Sugar free/ no added sugar	26	7·8
Healthy food	20	6·1
Provides essential nutrients	14	1·8
Physical activity depicted	40	12·3
Link to a special day/ event	64	19·6
Premium offers		
Competition	20	6·1
Not competition	20	6·1
Prompt to engage online	21	6·4
Level of engagement		
Median likes per post (p25 – p75) *n* 313^*^	646·8K	280·5K – 1·1M
Median comments per post (p25 – p75) *n* 323^*^	2·6K	1·1K – 5·0K
Persuasive appeal^†^		
Taste	68	20·9
Link to sports or entertainment event	35	10·7
Energy	35	10·7
New product information	31	9·5
Fun	24	7·4
Health/nutrition	23	7·1
Romance/sex	22	6·7
Premium/contest	13	4·0
Offers choices/options	13	4·0
Enjoyment/satisfaction	12	3·7
Humour	9	2·8
Holiday, travel or adventure	9	2·8
Corporate information	8	2·5
Friendship	5	1·5

* Comments were disabled, and the total number of likes was not available for certain posts.

† Presuasive appeals with the frequency of <1 % are not reported here.

#### Characteristics of advertisement

All posts included the brand name, while the product name was present in 89·6 % of the posts. Most of the posts featured F&B brands (89 %), and 11 % of the posts featured food retail enterprises. Twenty-nine posts featured more than one product, and thirty-three posts advertised brands while not showing any product.

#### Marketing techniques

Videos were used in 65·3 % of the posts, and 34·7 % of posts included still images. Half of the posts had music or added sound features. In almost all posts, the celebrities and influencers used the brand logo (94·2 %) and tagged the brand (96·9 %) as a symbol of association with the brand in advertisements. 84 % of the posts showed the image of the packaging, and 59·5 % of the posts showed the image of the product itself. 40 % of the posts did not explicitly mention the reason for featuring the product. 40 % mentioned it was a paid promotion, and 20 % of the posts were made by influencers who owned the F&B brand. The presence of other celebrities or influencers in the same post was found in 19·3 % of the posts. Health washing was present in 81 (24·8 %) posts. The most used health claim was ‘sugar-free or no added sugar’ (7·8 %), followed by ‘healthy food’ (6·1 %) and ‘provides essential nutrients’ (1·8 %). The prompt to engage with the post online by liking, commenting, sharing, tagging, following or posting pictures/videos (6·4 %) was not found to be frequent. Link to a special day or event was found in almost 19·6 % of the posts.

#### Level of engagement

The median number of likes per post was 646·8K, and the median number of comments was 2·6K, suggesting the level of online user engagement/interaction with the F&B advertisement in the sampled posts.

#### Persuasive appeal

The most frequently used persuasive appeals in the F&B advertisements by celebrities and influencers on Instagram in this study were taste, link to sports or entertainment events, energy, new product information, fun, health/nutrition and romance/sex (Table 4).

**Fig. 2:**
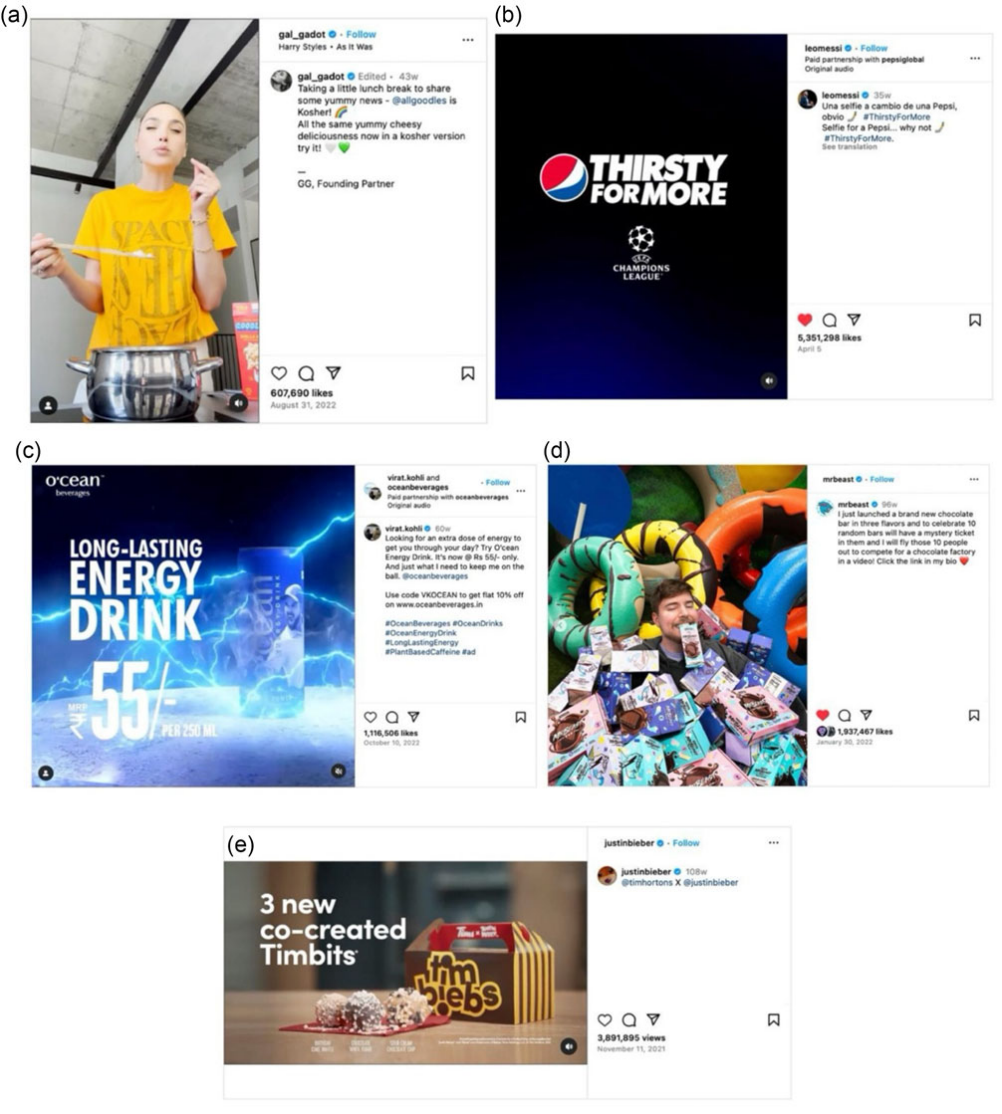
Examples of persuasive appeals. (a) Taste as a persuasive appeal. (*Source*: https://www.instagram.com/reel/Ch5CpNvDSPm/?igsh=MWludGd1Y3prZzlocw==); (b) Link to an event as a persuasive appeal. (*Source*: https://www.instagram.com/reel/CqpuojyLhzb/?igsh=cm11djc5NGdtYzJh); (c) Energy as a persuasive appeal. (*Source*: https://www.instagram.com/reel/CjhVOEqgH50/?igsh=MXVzYXRpbnZoeTRlZw==); (d) Fun as a persuasive appeal. (*Source*: https://www.instagram.com/p/CZVAueAP-U3/?igsh=dHEwZ3VjajRvcm5k); (e) New product information as a persuasive appeal. (*Source*: https://www.instagram.com/tv/CWHDfowBrvV/?igsh=OG12c3MyMTl3MHk2).

Taste was the most frequently used persuasive appeal in this study (20·9 %). The words ‘delicious,’ ‘yummy’ and ‘cheesy’ in the post shown in Fig. 2(a) were used by celebrities and influencers to express the appeal of the food product being advertised to have good taste. They also used hand gestures and facial expressions (as shown in Fig. 2(a)) to deliver the same message and make the advertisement appealing to adolescents. This post is also a typical example of a food advertisement by influencers who own the brand they are promoting. They use words like ‘founding partner’ or ‘founder’ in the caption to explicitly indicate their ownership.

The second most widely used appeal was the link to sports or entertainment events (10·7 %). Figure 2(b) shows a post from the Instagram account of an infamous footballer, and the link to the ‘UEFA Champions League’ football event was shown at the end of the most-liked video post of this research. Almost all the posts linking to the sports event were made by the sports person, and the brands featured in those posts were ‘PepsiCo’ and ‘Lays’. In another post by the same sportsperson shown in Fig. 2(b), the slogan ‘Lays and football better together’ was used to deliver the same meaning.

Energy was an equally popular appeal as the link to events in this study (10·7 %), used mainly in the posts featuring energy drinks. Figure 2(c) shows a regular example of such posts. Direct use of words like ‘long-lasting energy’ by an infamous cricketer in his post easily conveys the meaning and purpose of the product. Other commonly used words in energy drink advertisements included ‘power,’ ‘stamina’ and ‘strength.’ Creative visualisation techniques to show bursts of energy were also found commonly in such posts. Additionally, 12·3 % of posts featured physical activity. Such posts often depicted not only physical activity but also adventure, athletic endeavours and stunt performances. Most of the posts featuring energy drinks were made by sports persons, but entertainment celebrities, especially males with athletic endeavours, were also found to be posting about them.

Another persuasive appeal identified in this research was the new product information (9·5 %). Figure 2(e) represents an example of such posts. This post features three new products of the food retail enterprise Tim Hortons. Such posts often included details about the launch date of the new products. This post is also an illustrative example of the collaboration between influencers and brands. The explicit indication by the word ‘co-created’ shows the collaboration between Tim Horton and the influencer.

The fun associated with the product (7·4 %) was another frequently used persuasive appeal in this study. Figure 2(d) shows a typical example of a fun depiction in a post by a popular social media influencer. It conveys the message that this product will bring out the fun side. Facial expressions of the influencer, the setting of the products and the props coerce the fun appeal of the product in this post, which makes it appealing to adolescents. Dancing and upbeat music were also found to be used to express the fun after consuming the product in other posts.

## Discussion

This research is the first to analyse the contents of F&B advertisements through user-generated content by celebrities and influencers on Instagram. Entertainment celebrities and sportspersons on Instagram post content not only related to their interests but also to promote F&B on their followers’ feeds. Half of the posts explicitly indicated paid partnership, whereas half of the posts did not indicate the reason for featuring the product. Previous studies suggest that not mentioning the paid endorsement results in the content appearing as a peer recommendation rather than an advertisement^(9)^, which is not only misleading but also potentially harmful to teenagers. Evidence from a recent meta-analysis shows that exposure to unhealthy F&B marketing featuring celebrities or influencers significantly increases the consumption of unhealthy F&B among children and adolescents^(34)^, raising concerns about the health impact of such advertisements.

This study highlights the widespread prevalence of the marketing of F&Bs on Instagram through celebrities and influencers with massive followings ranging from 15 to 500 million. With 89·2 % of posts promoting unhealthy products, this raises concerns about the potential exposure of adolescents to such marketing, given Instagram’s popularity among teens. This aligns with findings from a previous research in Mexico that analysed posts on brand pages. They found that almost all products marketed by the brand pages exceed the threshold of critical nutrients^(31)^. Also, in the Philippines, a similar pattern was observed, where 99 % of the advertised F&Bs on the brand pages on Instagram were not permitted to be marketed according to the WHO criteria^(35)^. A significant portion of these advertisements promoting sugar-sweetened beverages and energy drinks is particularly concerning^(31,36)^. These products are not only high in added sugar but also contain non-nutritive sweeteners and stimulants, which pose considerable health risks^(37,38)^ and are therefore prohibited from being marketed to children and adolescents^(33)^. The overwhelming presence of such products may normalise the consumption of unhealthy F&B among younger audiences, making them susceptible to health risks. Furthermore, the vast reach of the influencers amplifies the marketing message, as adolescents are more likely to emulate the behaviour of those they follow^(22)^.

It was observed that celebrities and influencers frequently use brand logos in their posts, and it was present in 94 % of the posts in this study. Brand logos increase the recognition of the brand and the product and can generate lifelong brand loyalty among adolescents, making it the most important marketing strategy for the industry^(39)^. Apart from logos, the brand was also tagged in almost all posts, indicating the association of celebrities and influencers with the industry. Interestingly, images of product packaging (84 %) appeared more frequently than the image of the product itself (59·5 %). This suggests that packaging design may be utilised to strengthen brand identity, fostering a sense of familiarity and recognition. Contrary to the current study, previous researchers found almost equal frequency of the image of packaging and the product on Instagram brand pages^(31)^. This difference may indicate that influencer marketing employs distinct strategies as compared with corporate advertisements, potentially making advertisements more engaging and persuasive to young audiences.

Various other engagement techniques are also used by celebrities in social media posts to make them more interactive. A Facebook case study in New Zealand revealed interactive food marketing through frequent requests to like, comment or share the posts on the brand pages^(40)^. Although previous research has reported a high prevalence of prompts to engage online with the post^(31)^. But in the present study, the cues to engage online were only present in 6·4 % of the posts. This could be attributed to the substantial fan base of the celebrities and influencers because this study captured food advertisements by the top Instagram accounts, which already have a large number of followers and may not find the need to prompt active online engagement with their posts or accounts.

The persuasive appeal used most frequently in this study was taste (20·9 %). Influencers used both verbal and non-verbal gestures to convey the meaning of the taste of the food. Similar findings were reported in a previous study, where the word ‘Frosties taste gr-r-reat’ was displayed on television with the same voiceover for the advertisement of breakfast cereals^(41)^. Energy (10·7 %) was another recurring appeal in the present research, as energy drinks were the second largest advertised category of food (20 %). The use of adventure and stunt performance for the depiction of energy was commonly used by sportspersons or athletes. This aligns with the previous research demonstrating the use of sportspersons and the display of high energy and fiery adventure in the Instagram posts of the Monster energy drink^(42)^.

The link to the sports events was another widely used persuasive marketing appeal observed in the current study. Since the data for this research spanned from 2021 to 2023, encompassing the UEFA Champions League in 2021 and the FIFA World Cup in 2022, these events likely contributed to the frequent use of links to sports events in the posts. Therefore, it may not be representative of the marketing strategies used by Instagram influencers outside such sports events. Further research on the variation in marketing strategy on and off sports seasons will shed better light on this matter.

In the current study, 89·2 % of the advertised F&B products were found to be not permitted to advertise to adolescents. Exposure to food marketing by influencers has been associated with increased energy intake^(34,43)^, which is among the leading contributors to weight gain and obesity^(44)^. Although by policy, Instagram does not allow children to have accounts on their platform, previous statistics have shown the opposite^(15,45)^. Hence, the children are also not adequately protected from the potential exposure to unhealthy food marketing on social media. Instagram does not have any strategy or policy to tackle this issue of growing global concern. Only a few countries have statutory laws to regulate food marketing on the Internet^(46,47)^, and the countries that have laws forbidding digital marketing of unhealthy F&B do not encompass marketing directed towards adolescents and influencers marketing as third-party or de facto marketers^(48)^. Since the implementation and monitoring of public policies in the domain of online food marketing is challenging due to cross-border marketing, it is recommended that digital social media platforms design strategies for responsible F&B marketing to teens and ensure comprehensive compliance and implementation.

### Limitations

The main limitation of this research is that it studied the potential exposure of food marketing to adolescents through celebrities on Instagram, not the real-time exposure, and we do not know if these celebrities and influencers appeal to adolescents. Second, this research only studied F&B marketing through Instagram, just one of several popular social media platforms. Although the influencer accounts included in our sample were selected based on popularity and engagement metrics, we did not have access to specific demographic data about followers. As such, we cannot verify the extent to which the audiences for these accounts were adolescents. Our assumption that adolescents are likely among the audiences of high-engagement influencers was informed by global patterns of adolescent social media use, but remains a limitation.

While the WHO CLICK framework offers a standardised and widely used tool for monitoring food marketing to children and adolescents^(25)^, we found that it does not provide guidance on determining the adolescent specificity of coding categories. We recommend refining the framework to better distinguish age-relevant content could strengthen its utility and precision to help capture marketing that is most likely to affect adolescent behaviour for future studies.

However, given the high use of Instagram and the feasibility of collecting the data, a focus on one social media serves as a starting point for this line of research.

## Conclusion

This study provides evidence of the widespread presence of persuasive marketing of unhealthy F&B on Instagram, suggesting that adolescents may be exposed to such content, given the platform’s popularity among this age group. Policies mandating the clear disclosure for paid promotions in the user-generated content on social media are urgently needed to reduce adolescent’s exposure to covert F&B marketing. Without enforced regulations that limit such posts, it is reasonable to conclude that adolescents will continue to be exposed to more content that encourages unhealthy eating behaviours and that contributes to poor health and health system burden in the long run. Therefore, adolescents and their parents need to be better educated and aware of the persuasive appeals used in marketing F&B online. Such awareness may not make them less vulnerable to their effects, but better education about the effects and how not to fall victim to them could help reduce the power of such appeals and techniques. Further research is certainly warranted on the impact of these posts on actual behaviours, educational activities designed to empower adolescents and the impact of social media posting regulations on the exposure and power of F&B marketing. This research examined what was posted on Instagram. The next step could be examining real-time exposure to posts using screen captures and eye-tracking methods. This could generate better evidence regarding the influence these posts have and the subsequent development of relevant policy.
